# Correlation between Social Determinants of Health and Women’s Empowerment in Reproductive Decision-Making among Iranian Women

**DOI:** 10.5539/gjhs.v8n9p312

**Published:** 2016-01-31

**Authors:** Zahra Kiani, Masuomeh Simbar, Mahrokh Dolatian, Farid Zayeri

**Affiliations:** 1 Department of Midwifery and Reproductive Health, Student Research Office, School of Nursing and Midwifery, Shahid Beheshti University of Medical Sciences, Tehran, Iran; 2 Department of Midwifery and Reproductive Health, School of Nursing and Midwifery, Reproductive Endocrinology Research Center, Research Institute for Endocrine Sciences Shahid Beheshti University of Medical Sciences, Tehran, Iran; 3 Department of Midwifery and Reproductive Health, School of Nursing and Midwifery, Shahid Beheshti University of Medical Sciences, Tehran, Iran; 4 Proteomics Research Center and Department of Biostatistics, Faculty of Paramedical Sciences, Shahid Beheshti University of Medical Sciences, Tehran, Iran

**Keywords:** women’s empowerment, reproductive health, social determinants of health, reproductive decision making, empowerment, self-esteem

## Abstract

**Background and Objectives:**

Women empowerment is one of millennium development goals which is effective on fertility, population’s stability and wellbeing. The influence of social determinants of health (SDH) on women empowerment is documented, however the correlation between SDH and women’s empowerment in fertility has not been figured out yet. This study was conducted to assess correlation between social determinants of health and women’s empowerment in reproductive decisions.

**Material and Methods:**

This was a descriptive-correlation study on 400 women who attended health centers affiliated to Shahid Beheshti University of Medical Sciences Tehran-Iran. Four hundred women were recruited using multistage cluster sampling method. The tools for data collection were 6 questionnaires including; 1) socio-demographic characteristics 2) women’s empowerment in reproductive decision-making, 3) perceived social support, 4) self-esteem, 5) marital satisfaction, 6) access to health services. Data were analyzed by SPSS-17 and using Pearson and Spearman correlation tests.

**Results:**

Results showed 82.54 ± 14.00 (Mean±SD) of total score 152 of women’s empowerment in reproductive decision making. All structural and intermediate variables were correlated with women’s empowerment in reproductive decisions. The highest correlations were demonstrated between education (among structural determinants; r= 0.44, P< 0.001), and Self-esteem (among intermediate determinants; r= 0.34, P< 0.001) with women’s empowerment in fertility decision making.

**Conclusion:**

Social determinants of health have a significant correlation with women’s empowerment in reproductive decision-making.

## 1. Introduction

Women’s empowerment means to empower women in order to take independent decisions (Qureshi, & Shaikh, 2007), which can change women’s lives (Nayak, & Mahanta, 2009). Basically, empowerment can develop individuals’ ability to make their principle life choices (Kabeer, 2005). Empowered women demand equal access to health services and this may, in turn, lead to improved reproductive health (WHO Department of Gender, 2008).

According to the World Health Organization, approximately 800 women lost their lives from pregnancy and childbirth complications each day and 289,000 maternal deaths occurred in 2013 (WHO, 2014). For every maternal death, at least 30 women suffer from debilitating diseases and injuries (Yacobson, Christopherson, & Michaelides, 2012). International Conference on Population and Development (ICPD) in Cairo considers women’s empowerment as the basis for development plans (M. Campbell, SahinHodoglugil, & Potts, 2006) as it is one of the most effective factors for deceasing maternal and infant mortality and morbidity. The main core of women’s empowerment is not only having equal facilities (such as education) and access to resources (such as land and occupation), but having also the power for decision making and using these rights and facilities (UNDP, 2007). Women, especially in developing countries, suffer from problems related to reproductive system and sexual behaviors (Froozanfar, Majlessi, Rahimi, & Pourreza, 2012). Sexual discrimination in society affects women’s health (Wusu, & Isiugo-Abanihe, 2004), and leads to social and economic changes, which affect health and lead to gender gap (Marmot, Allen, Bell, Bloomer & Goldblatt, 2012). In the Cairo Conference, the fourth principle emphasizes on women’s empowerment, gender equity and equality and eliminating violence against women (ICPD, 1994). In fact, Cairo Conference simultaneously considered the emphasis on women’s empowerment in reduction of gender inequality and promotion of health and economic resources (Williams, 2005).

Social determinants of health mean conditions in which people are born, grow, live and work (Marmot et al., 2012). According to the World Health Organization model on the social determinants of health, two groups of determinants have been defined: structural including gender, income, education, occupation and ethnicity, and intermediate determinants including material circumstances (i.e. home and work), psychosocial factors (i.e. psychosocial stresses) and behavioral factors (i.e. smoking) (Mackenbach, & Bakker, 2002).

However, most studies have investigated factors as the solutions to achieve the objectives of family planning, only within the health system, while family planning infrastructure should be considered in any social and cultural environment (Stephenson, & Hennink, 2004). Social status and cultural norms could be significant barriers to women’s empowerment (CARE, 2010). Underpowered women (in terms of education and occupation) have more unmet needs for family planning (Motlaq, Eslami, Yazdanpanah, & Nakhaee, 2013). These women do not use effective methods of contraception due to improper information and lack of support from the husband and community as well as low access to services (Pakseresht, Mirnaghjoo, Kazamnejad, & Vazifeshenas, 2005). Notably, providing voluntary access to family planning services is effective not only in improving reproductive outcomes, but also on health, education and economy (Ahmed, Li, Liu, & Tsui, 2012; Canning & Schultz, 2012; Cleland, Conde-Agudelo, Peterson, Ross, & Tsui, 2012).

Women’s independence and their power for making decision increases their reproductive control, attitudes and ability for childbearing planning (Sujatha, & Reddy, 2009). In the other hand, husbands’ opposition and their negative attitudes towards family planning are the main reasons for the unmet needs (Motlaq et al., 2013).

## 2. Objectives

Although there is some information on relation between women’s empowerment and social determinants of health, no study has been conducted so far on the correlation between social determinants of health and women’s empowerment in reproductive decision-making, hence this study was performed to assess correlation of social determinants of health with women’s empowerment in reproductive decision-making.

## 3. Materials and Methods

### 3.1 Study Population and Area

This descriptive correlational study was conducted on 400 women selected by multistage cluster sampling among those attending health centers of Shahid Beheshti University of Medical Sciences Tehran-Iran. All health centers affiliated to University of Medical Sciences were considered as clusters. In the first stage, all health centers were listed, coded and then 12 centers were selected by simple random method from 54 centers. Then the sample size in each center was calculated by quota proportionate to the population covered by each center, and finally sampling was conducted at health centers by convenience method.

### 3.2 Sample Size

Due to lack of information on women’s empowerment in reproductive decision-making, the sample size was determined by a pilot study on 40 women with inclusion criteria: age range of 15-49 years, no history of depression, good mental health and appropriate communication to fill out the questionnaire, married and currently living with their husbands and their husbands’ only wife, at least one child and not being pregnant, literate, living in Tehran for more than one year, no history of infertility and relevant treatments, Iranian nationality and Muslim. The sample size was calculated (400).

### 3.3 Ethical Approval

This study was approved by the Ethics Committee of Shahid Beheshti University of Medical Sciences, with code 1392-1-86-12716. Written permission was issued by Shahid Beheshti University of Medical Sciences for sample recruitment in the health centers. The study objectives were explained to the participants as well as they were ensured about the confidentiality of their information. Informed consent was obtained from them and then they filled out the questionnaires in a private setting.

### 3.4 Questionnaire Measures

Data collection instruments were questionnaires on structural social determinants of health (socioeconomic status: education, occupation and asset indicator) and intermediate social determinants of health (psychosocial factors: self-esteem, social support and marital satisfaction, and health systems: access to health services), demographic characteristics and women’s empowerment in reproductive decision–making.

#### 3.4.1 Demographic Characteristics

The researcher-made questionnaire for demographic characteristics and reproductive profile included 9 items on age: the number of deliveries, the number of abortions, age at marriage, age at first childbirth, children’s gender, the number of children, unwanted pregnancy and income.

#### 3.4.2 Socio-Economic Questionnaire

The questionnaire about structural social determinants of health included socio-economic questionnaire containing 12 items designed to assess men’s and women’s education, income, women’s and their husband’s occupation. The number of schooling years was used to investigate education, the asset indicator including twelve economic variables (vacuum cleaner, separate kitchen, computer, washing machine, bathroom, freezer, dishwasher, private car (not for working), mobile, color TV, various types of video and telephone) was used to assess income (Baheiraei, Hamzehgardeshi, Mohammadi, Nedjat & Mohammadi, 2013) and was calculated as percentage, and women’s and men’s employment status was asked by open-ended questions in the questionnaire and was classified based on occupation classification (Rose, O’Reilly, & Britain, 1998). This category has 17 main categories, each with subcategories that totally define 30 occupational categories, L17 was coded as 1 by default based on which other categories were coded and finally, occupation were assigned to 30 categories.

#### 3.4.3 Perceived Social Support Questionnaire

Multidimensional Scale of Perceived Social Support (MSPSS) by (Zimet, Dahlem, Zimet, & Farley, 1988) is a 12-item instrument to assess perceived social support from three sources of family, friends and significant others. In this test, the scores of 1 to 7 are respectively given to ‘totally disagree’, ‘disagree’, ‘almost disagree’, ‘no idea’, ‘almost agree’, ‘agree’, and ‘totally agree’. The scores of 12-48 show low social support, scores of 49-68 show moderate social support and scores of 69-84 show a high level of social support. Validity of Multidimensional Scale of Perceived Social Support questionnaire was reported appropriate. The questionnaire’s reliability was reported between 0.89 and 0.91 by Cronbach’s alpha for subscales (Canty-Mitchell & Zimet 2000; Zimet et al., 1988) and 0.92 for the entire questionnaire and intra-cluster correlation was reported 0.89 (Dolatian et al., 2014; Mirabzadeh et al., 2013).

#### 3.4.4 Rosenberg Self-Esteem Scale

Rosenberg self-esteem scale is a standard questionnaire including 10 items with four options of totally agree, agree, disagree, and totally disagree with scores from 1 to 4 and the total score is the sum of all scores given to 10 items. It has been reported that the concurrent, predictive and construct validities are appropriate. The reliability was reported 0.85 and 0.88 by test-retest and the internal consistency was excellent (Rosenberg, 1965).

#### 3.4.5 Marital Satisfaction Questionnaire

Marital satisfaction questionnaire was developed by Walter W. Hudson in 1992 with the aim to measure marital problems. It has 25 items, and 2 cut-off points: scores greater than 30 indicate significant clinical problems and scores greater than 70 suggest severe stress. Questionnaire of Marital Satisfaction is valid with appropriate construct and concurrent validity (Torkan, & Moulavi, 2009). Reliability has been reported excellent by test-retest (Golestani, Tavakoli Manzeri, & Tavakoli Manzeri, 2012; Lee, Yip, Leung, & Chung, 2004), and the internal consistency is excellent too (Lee et al., 2004).

#### 3.4.6 Access to Health Services Questionnaire

The researcher-made questionnaire for access to health services includes 9 items with four-point Likert scale (score of 0 for no effect, 1 for little effect, 2 for moderate effect and 3 for high effect). Finally, scores are added up and considered as the score of access to health services. In this study, test-retest was used to investigate the reliability of access to health services questionnaire. The correlation of scores of access to health services in test-retest of the questionnaire was 0.85.

#### 3.4.7 Women’s Empowerment in Reproductive Decision–Making Questionnaire

Women’s empowerment in reproductive decision–making (WERD) questionnaire (Kohan, Simbar, & Taleghani, 2012) measures women power in making decision on their reproduction. This questionnaire includes 38 items with five-option Likert scale (the score of 4 for totally agree, 3 for agree, 2 for no idea, 1 for disagree, and 0 for totally disagree) in four dimensions: cultural (11 items), individual and family (10 items), social (9 items) and family planning (8 items). Then the total score range of WERD is between 0-152.

The content validity rate (CVR=0.62) and content validity index (CVI) were calculated for WERD-questionnaire. The content validity rate was developed by Lawsh (Lawshe, 1975). Content validity index (CVI) was calculated based on the criterion of Waltz and Bausell (Yaghmaei, 2003), and the relevance, clarity and simplicity of each item was also determined. First, the relevance of all items was investigated and the mean of 0.96 was obtained and then the mean of simplicity and clarity was reviewed and obtained as 0.93 and 0.94, respectively.

For assessing the reliability of women’s empowerment reproductive decision making instrument, stability was determined by test-retest and internal consistency was determined by Cronbach’s alpha. In the test-retest method, the correlation of 0.77 was obtained. Cronbach’s alpha coefficient was found 0.76, 0.71, 0.73, 0.69 and 0.72 for cultural, social, individual–family, family planning domains and the entire questionnaire, respectively. Generally, alpha coefficient of 0.70 or higher is acceptable to estimate the reliability and the lower values are considered low reliability (Di Lorio, 2005).

Data were statistically analyzed by SPSS-17, using Pearson and Spearman correlation tests.

## 4. Results

In total, 400 women completed questionnaires from April to June 2014 at health centers affiliated to Shahid Beheshti University of Medical Sciences Tehran-Iran. Their demographic characteristics suggest that the subjects’ mean age was 31.10 ± 6.50 years, most of them had one child (48.8%), had gotten married when they were 18-28 years (76.5%), and had their first child during the same age range (85.5%).

Information on Women’s empowerment in reproductive decisions implied that the mean score of women’s empowerment in reproductive decision-making was 82.54 ± 14.00 of the total score of 152, on the other words, they obtained 54.3% of the total score. The highest score in WERD- questionnaire was in the cultural domain (63.2%) and the lowest score in WERD- questionnaire was in family planning (34.7%) (Table 1).

**Table 1:** Women’s empowerment in reproductive decision making by domains (n=400)

domains of Women empowerment in reproductive decisions	Mean	Standard deviation	Minimum-Maximum	The percentage of obtained scores
cultural	27.8	5.03	12-42	63.25%
social	18.68	4.51	6-32	51.9%
individual and family	24.93	7.06	7-40	62.3%
family planning	11.12	5.71	0-28	34.7%
(total score)	82.54	14.00	42-114	54.3%

The mean, standard deviation, lowest and highest Obtained scores for intermediate and structural determinants of health are shown in Table 2.

Information on women’s and husbands’ occupation classification as the variable of social structural determinant of health is based on occupation classification of Ross et al. (1998) which suggests that most women were in subcategory 1 as Not classifiable for other reasons (L17) and most men were in subcategory 11 as Semi-routine technical occupations (L12-3).

The correlation between variables and women’s empowerment in reproductive decision-making was assessed before performing structural equation modeling. All structural and intermediate variables were correlated with women’s empowerment in reproductive decisions, and the highest correlation was found with education among structural determinants (r= 0.44, P< 0.001) and with Self-esteem among intermediate determinants (r= 0.34, P< 0.001) (Table 3).

**Table 2:** Mean and standard deviation of scores by the structural and intermediate social determinants of health (n=400)

Variable	Mean	Range of obtainable scores	Minimum- Maximum
Women’s education (years)	11.40±3.30	-	2-19
Men’s education (years)	11.30±3.57	-	4-23
Asset indicator (%)	80.21±15.13	0-100	8-100
Self-esteem	29.04±3.30	10-40	17-40
Multidimensional Perceived Social Support	60.46±12.48	12-84	12-84
Marital satisfaction	44.43±14.46	0-100	17-85

**Table 3:** The correlation between structural and intermediate social determinants of health with women’s empowerment in reproductive decisions (n=400)

Variable	Women’s empowerment in reproductive decisions	Women’s education	Men’s education	Women’s occupation	Men’s occupation	Asset indicator	Self-esteem	Social support	Access to health services	Marital satisfaction
Women’s empowerment in reproductive decisions	1									
Women’s education	0.44**	1								
Men’s education	0.36**	0.69**	1							
Women’s occupation	0.22**	0.32**	0.28**	1						
Men’s occupation	0.29**	0.44**	0.57**	0.25**	1					
Asset indicator	0.39**	0.46**	0.44**	0.28**	0.36**	1				
Self-esteem	0.34**	0.31**	0.27**	0.18*	0.097*	0.24**	1			
Social support	0.32**	0.34**	0.29**	0.16*	0.17*	0.23**	0.37*	1		
Access to health services	0.23**	0.14*	0.12*	0.15*	0.18*	0.20**	0.14*	0.23**	1	
Marital satisfaction	-0.33**	-0.28**	-0.25**	-0.12*	-0.14*	-0.21**	-0.44**	-0.58**	-0.07	1

* All values are significant *P* < .05

** All values are significant P< 0.001

## 5. Discussion

This study investigated the correlation between social determinants of health and women’s empowerment in reproductive decisions in Iranian society for the first time. The results showed that women’s empowerment was 54.3%. (Chaudhry, & Nosheen, 2009) reported that women obtained 41% of the total score of power and this can be attributed to cultural differences. Their findings suggested that the lowest power level was in social and family planning domains. Women’s differences should be considered in providing health services (Creel, Sass, & Yinger, 2002) and reproductive health services should be directed towards a more comprehensive perspective to help women’s Empowerment in reproductive decision making (Gwatkin, 2009). Moreover, social norms act as a barrier to women’s empowerment (Chaudhry, & Nosheen, 2009), which needs to be overcome.

Our findings showed that structural and intermediate social determinants of health are correlated with women’s empowerment in reproductive decision making. According to the World Health Organization model, structural determinants are social determinants such as education, income and occupation (WHO, 2011) and indicate people’s socioeconomic status (Graham, 2004). This mechanism organizes health opportunities of social groups based on placing them in the hierarchy of power, authority and access to resources (Kunst, & Mackenbach, 1990; Muntaner, Borrell, Benach, Pasarín, & Fernandez, 2003). According to the Cairo conference, good economic conditions and gender equality are important facilitators for women’s empowerment in reproductive decisions in health and reproductive health, but these domains were attended to much less after the conference (Williams, 2005).

Finding of the present study showed a significant correlation between women’s empowerment in reproductive health decision making and women’s and men’s education. Education is one of the accelerating factors for women’s empowerment and not only will increase skills at individual level but also provide people the ability to communicate effectively with the outside world and use resources to achieve domestic objectives (Kabeer, 2005) and lead to better adoption of health messages (WHO, 2011).

Women’s and men’s occupation were correlated with women’s empowerment in reproductive health decision making in the present study. Occupation reflects independence, control, and social network. It reflects social status and may be associated with health outcomes for it provides higher special privileges such as easy access to better healthcare services, access to better education and housing for people with higher social status (WHO, 2011). Full and productive employment and decent work for all is a new objective in the Millennium Development Goals (MGDs) (Health & Organization, 2008). Working conditions also affect health and equity, (Muntaner, Anthony, Crum, & Eaton, 1995). (Pandey, & Singh, 2008) showed that employed women felt they were more empowered and superior in the community.

Results of the study showed a significant correlation between asset indicator and women’s empowerment in reproductive health decision making. In this study, we used the asset indicator instead of income. Income is one of the essential components of increasing women’s power (Haile, & Enqueselassie, 2006) and leads to growing self-confidence (WHO, 2011). Husband’s income is effective on women’s decision-making to receive services (Rahman, Abedin, & Kamruzzaman, 2008). Women with low incomes receive lower-quality health services (Becker, Koenig, Mi Kim, Cardona, & Sonenstein, 2007).

Intermediate determinants of health were also correlated with women’s empowerment in reproductive health decision making in our study. Intermediate determinants include Material Circumstances, psychosocial factors and behavioral and biological factors and health system. Socioeconomic and political domains affect intermediate determinants. Unequal distribution of intermediate determinants through socioeconomic status is considered as the primary mechanism for health inequality (WHO, 2011), although, girls’ and women’s education is a necessary prerequisite for empowerment, social and cultural norms are considered major barriers (CARE, 2010). Cultural and social values and norms have a major impact on women’s decision-making about reproductive issues (Haile, & Enqueselassie, 2006).

Marital satisfaction was also significantly correlated with women’s empowerment in reproductive health decision making. In a study by (D’Souza, Somayaji, & Subrahmanya Nairy, 2011) marital relationship was effective on reproductive health and women’s health. In a study by (Kariman, Simbar, Ahmadi, & Vedadhir, 2014) reported that marital satisfaction affected decision-making on having the first child. In a study by (Mullany, Hindin, & Becker, 2005) observed a relationship between women’s reproductive health and men’s involvement, which was an important strategy to achieve women’s empowerment. Marital relationships and good economic status results in women’s noticing their health needs, and reproductive health interventions should be planned with family and husband (Kaddour, Hafez, & Zurayk, 2005).

Finding of the study revealed a correlation between self-esteem and women’s empowerment in reproductive health decision making. Extensive research has revealed a relationship between self-esteem, emotions, adjustment and health (Goswick, & Jones, 1981; Taylor & Brown, 1988). Women’s independence is a key factor in achieving their reproductive desires (Woldemicael, 2007) and will lead to women’s empowerment in planning for childbearing (Sujatha, & Reddy, 2009).

Social support was also correlated with women’s empowerment in reproductive health decision making in the present study. Lack of social networks is one of the barriers to women’s receiving health services (Qureshi & Shaikh, 2006). Social support and relationships have a significant contribution to health. The practical and emotional social supports that people receive vary depending on socioeconomic status (Wilkinson, & Marmot, 2003). In the study (Kariman et al., 2014) social support affected decision-making on having the first child.

Results of the study showed a significant correlation between access to health services and women’s empowerment in reproductive health decision making, Factors of access to health services can be a barrier to equal care (W. Campbell, Sleath, & Bosco, 2002). Social empowerment strategies can increase social awareness of health and health care systems, strengthen health literacy and encourage measures related to health (Loewenson, 2003; Vega-Romero & Tovar, 2007).

The role of health system is applied particularly through access to health services. Furthermore, differences in access to health services will definitely affect health outcomes (WHO, 2011). Providing voluntary access to family planning services improves reproductive outcomes (Ahmed et al., 2012; Canning & Schultz, 2012; Cleland et al., 2012).

## 6. Conclusion

Women’s empowerment in reproductive is a multidimensional process, and family, society and women should be considered for empowerment in reproduction. Factors such as socioeconomic factors, health system and psychosocial factors are among social determinants of health that have a significant correlation with women’s empowerment in reproductive decision making.

**Limitation**

Considering some aspects of social determinants of health as the effective factors on women’s empowerment in reproductive decision making and conducting the study at the health centers affiliated to Shahid Beheshti University of Medical Sciences Tehran-Iran were the limitation of the study.
